# Supplementary material for: Recombination of X-ray-Generated Radical Ion Pairs in Alkane Solution Assembles Optically Inaccessible Exciplexes from a Series of Perfluorinated para-Oligophenylenes with N,N-Dimethylaniline
Source: Int J Mol Sci. 2023 Apr 20;24(8):7568. doi: 10.3390/ijms24087568 (PMC10142361; doi:10.3390/ijms24087568)
Supplement: Supplementary file 1 [file ijms-24-07568-s001.zip › ijms-2336352-supplementary.pdf]

Supporting Information for

Recombination of X-Ray Generated Radical Ion Pairs in Alkane Solution Assembles Optically Inaccessible Exciplexes from a Series of Perfluorinated *para*-Oligophenylenes with *N,N*-Dimethylaniline

P.V. Nikul'shin, R.G. Fedunov, L.V. Kuibida, A.M. Maksimov, E.M. Glebov, D.V. Stass

Contents:

1. Optical characterization of solvents used for optical and X-ray studies
2. Optical characterization of decafluorobiphenyl **1**
3. Optical characterization of perfluoro-*para*-terphenyl **2**
4. Optical characterization of perfluoro-*para*-quaterphenyl **3**
5. Optical characterization of perfluoro-*para*-quinquephenyl **4**
6. Chemical structures and atom numbering systems of **1**, **2** and DMA
7. Calculated/experimental absorption/emission spectra for DMA
8. Geometric and electronic structures of the needed states for **1**, **2** and DMA
9. Juxtaposition of calculated and experimental emission spectra for single molecule/exciplex for **1**, **2** and DMA

1. Solvents used for optical and X-ray studies

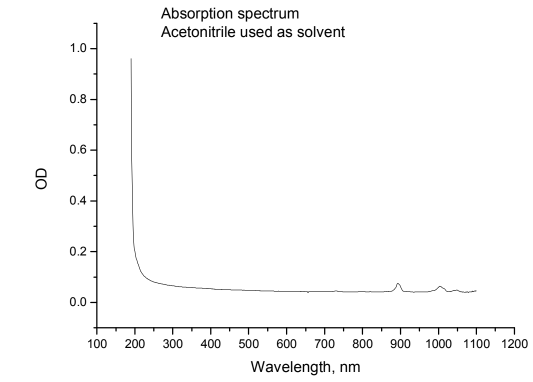

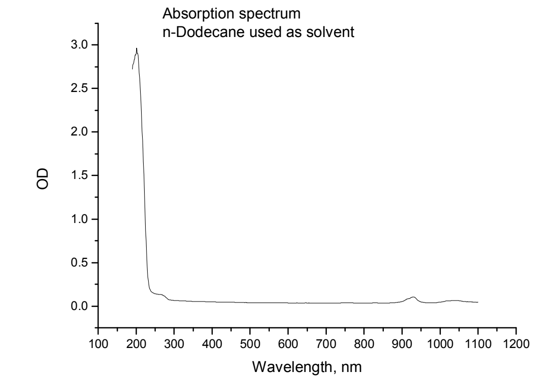

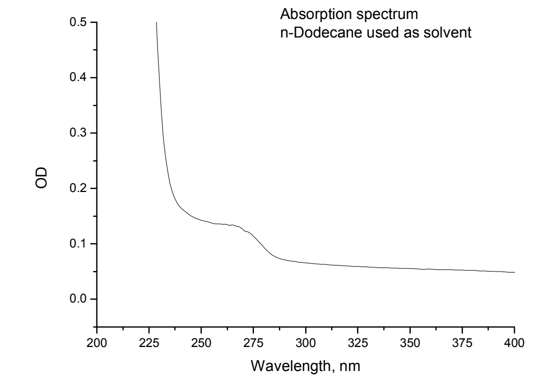

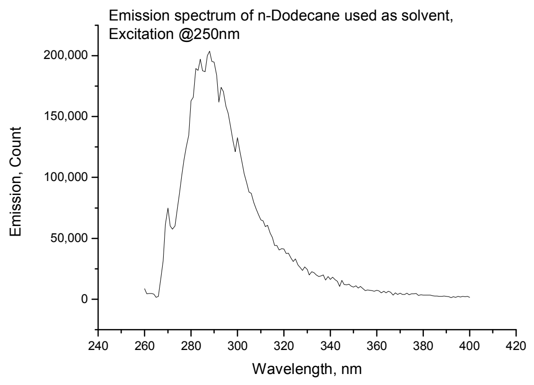

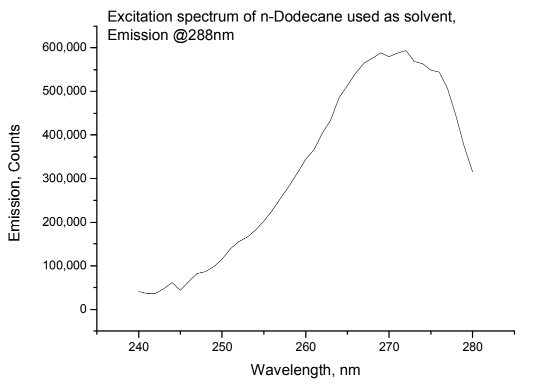

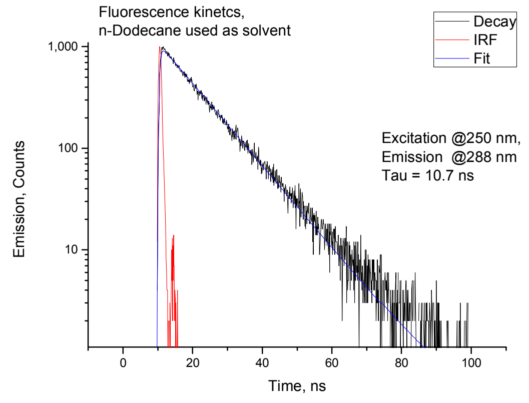

2. Decafluorobiphenyl **1**

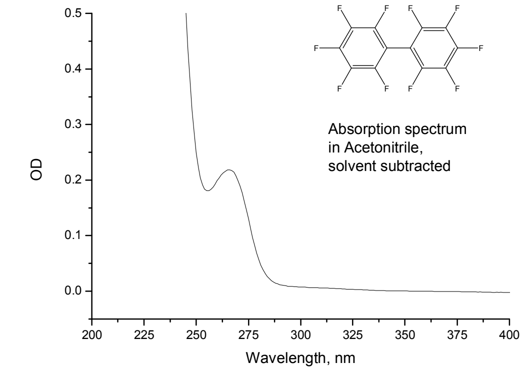

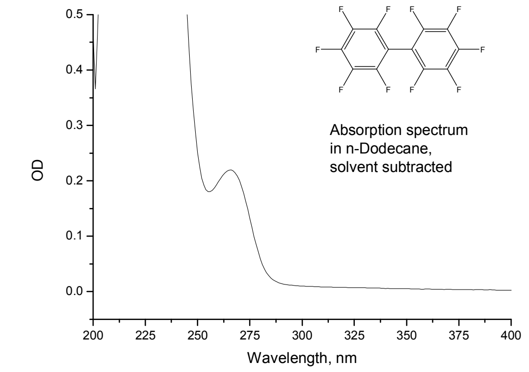

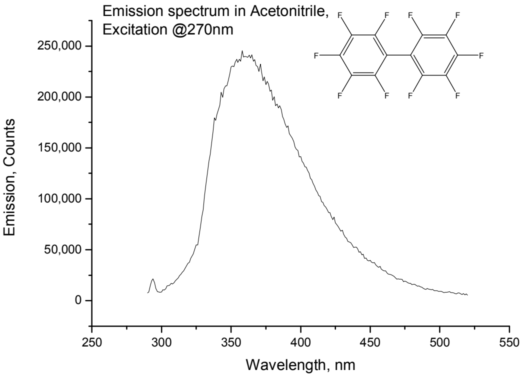

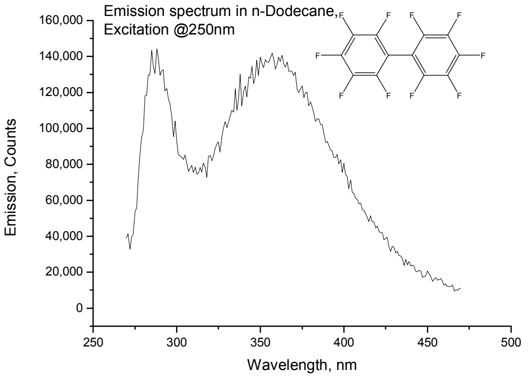

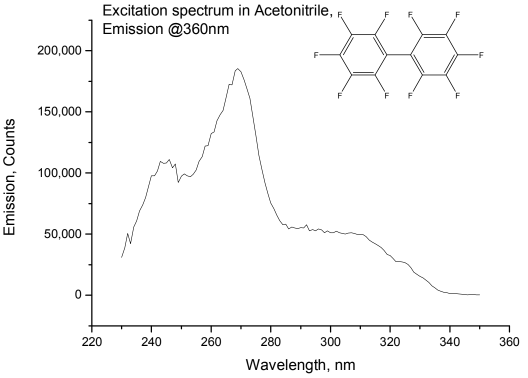

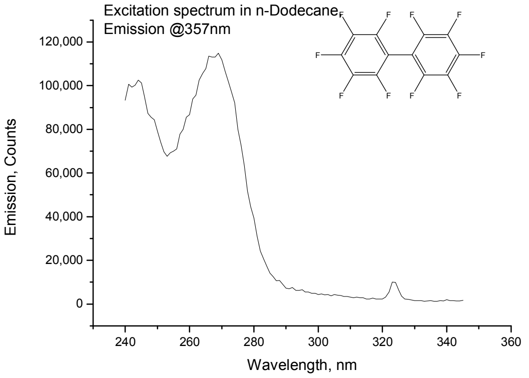

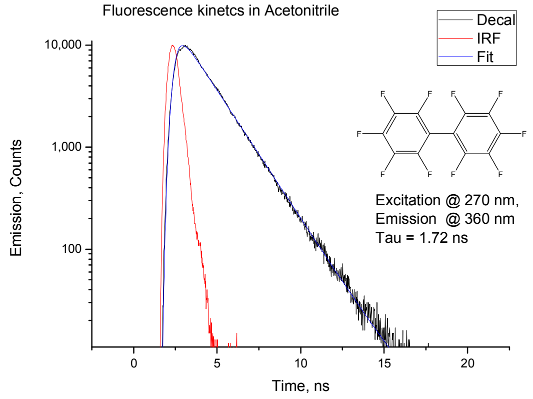

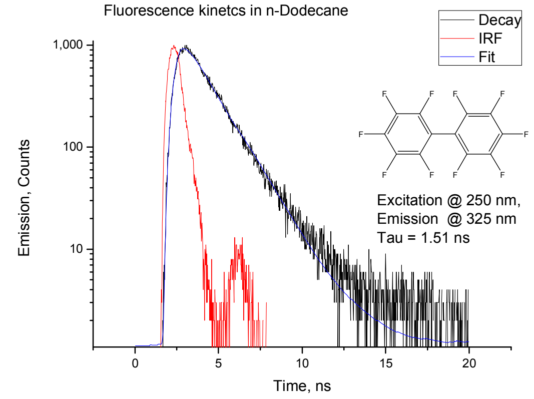

3. Perfluoro-*para*-terphenyl **2**

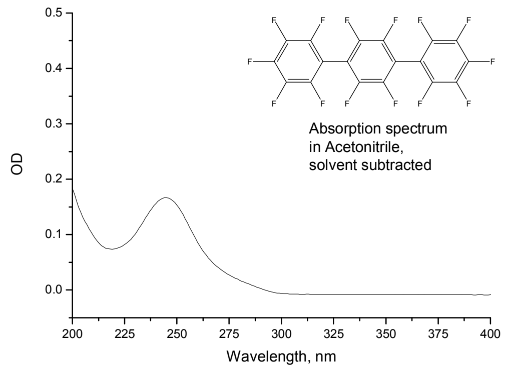

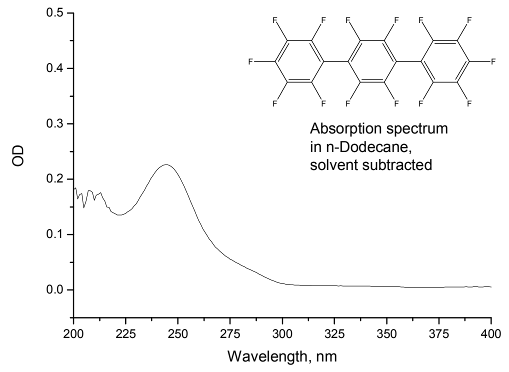

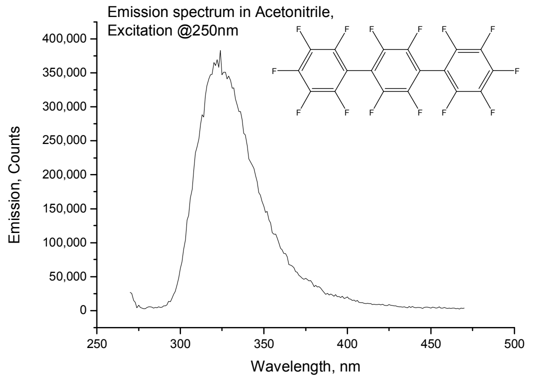

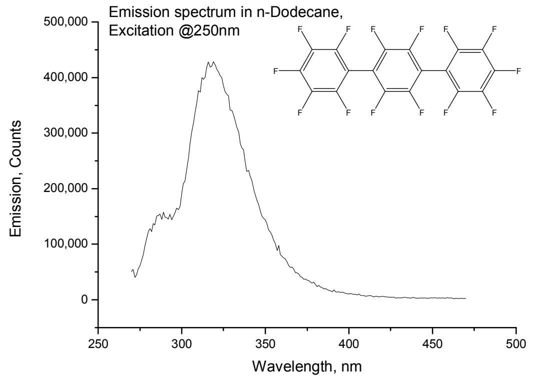

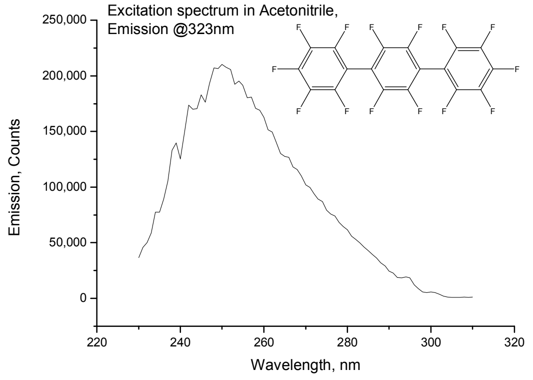

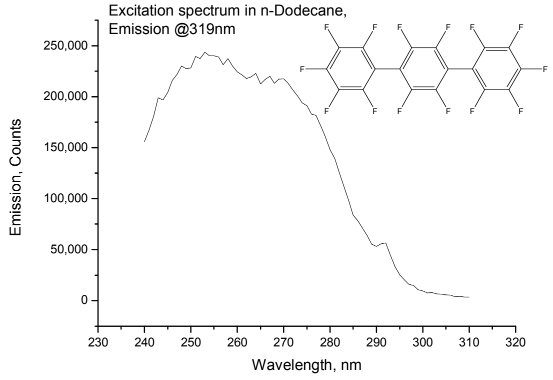

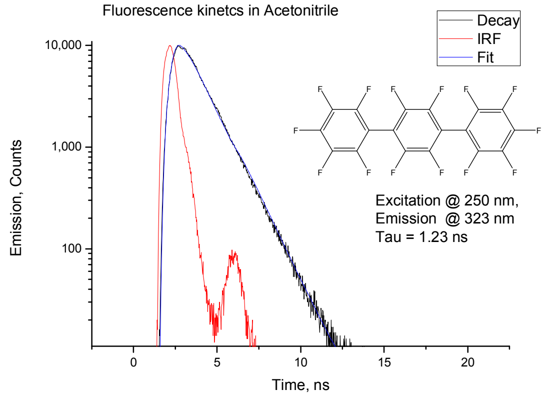

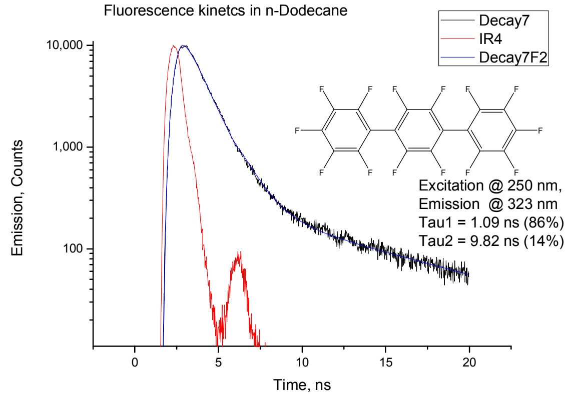

4. Perfluoro-*para*-quaterphenyl **3**

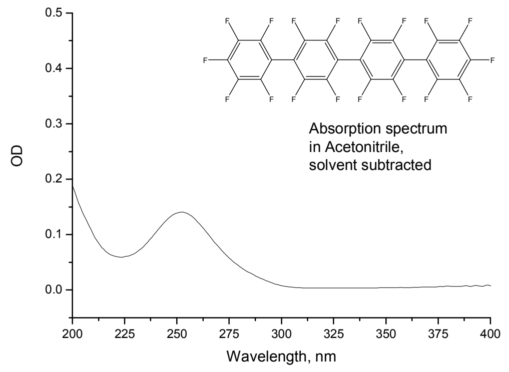

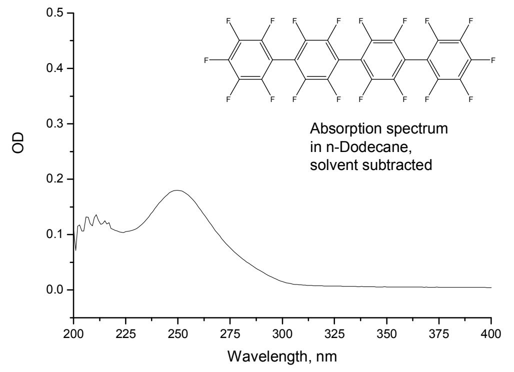

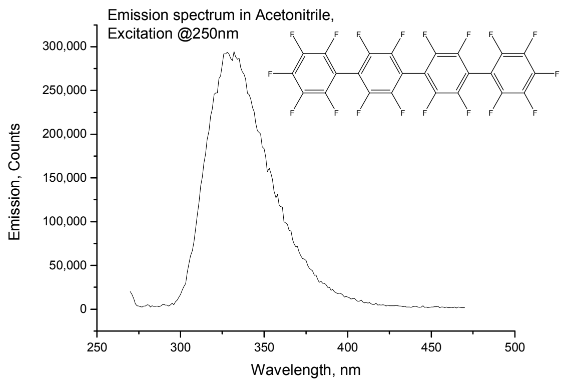

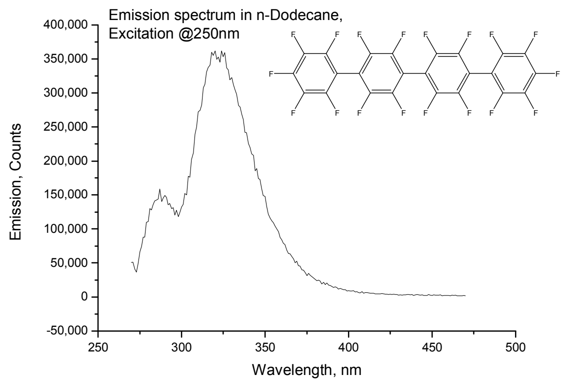

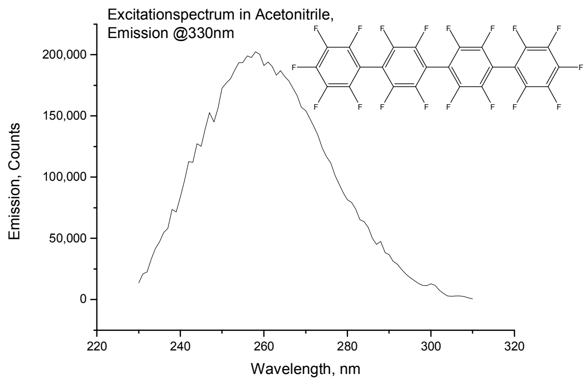

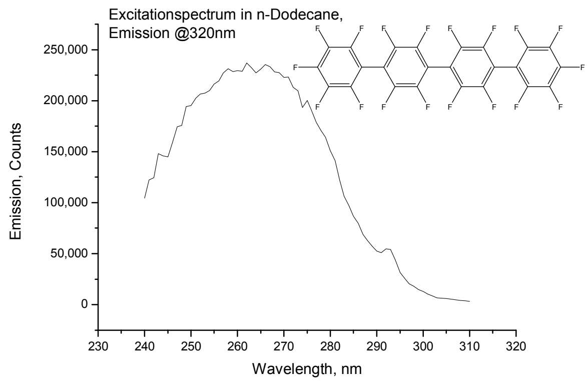

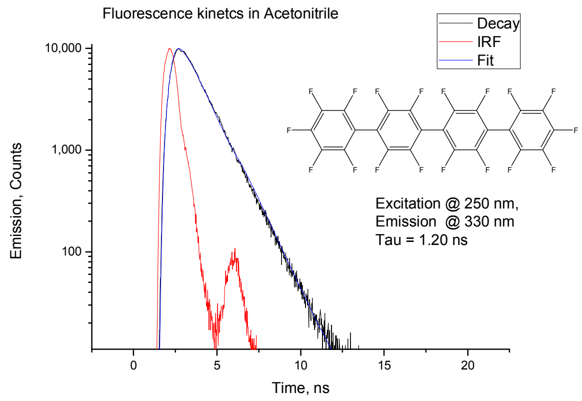

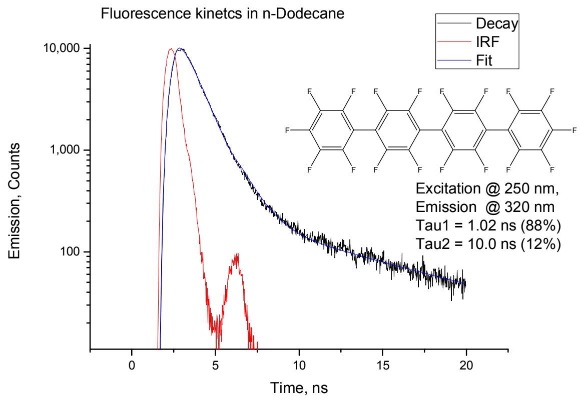

5. Perfluoro-*para*-quinquephenyl **4**

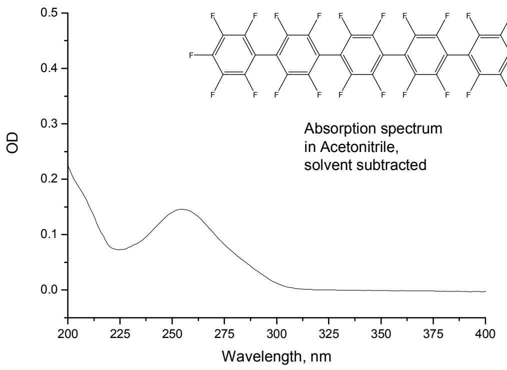

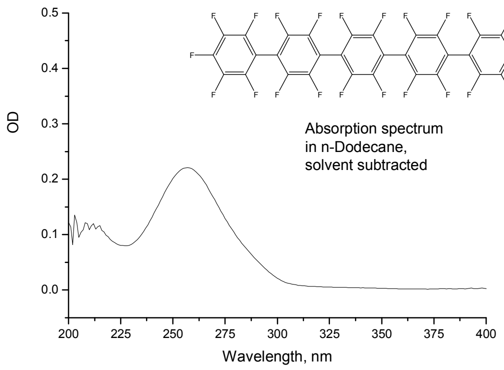

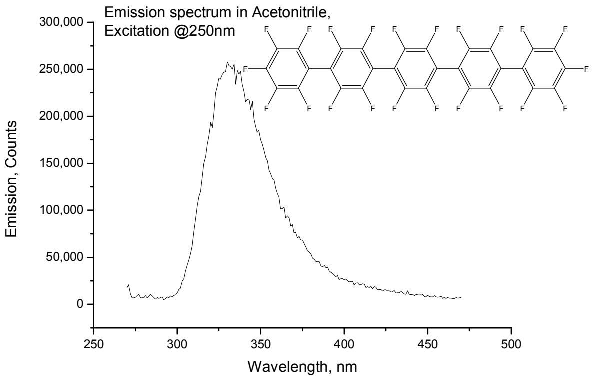

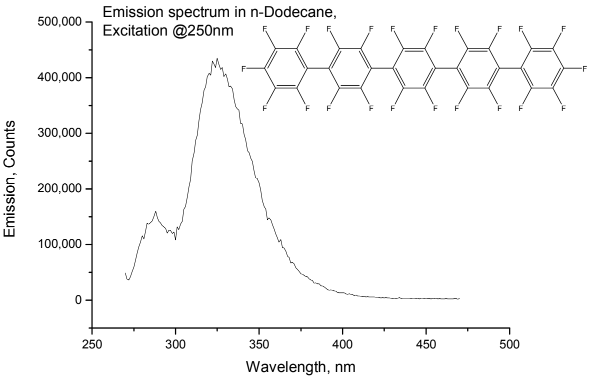

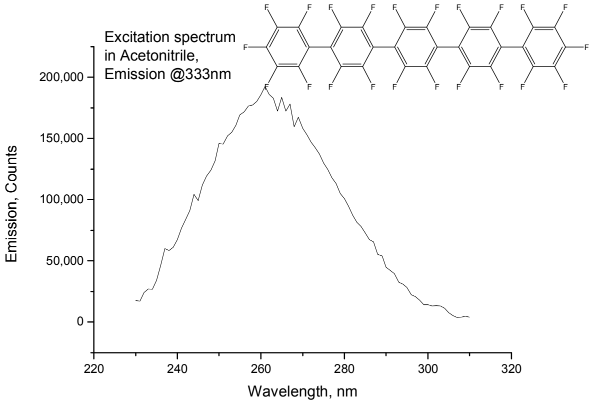

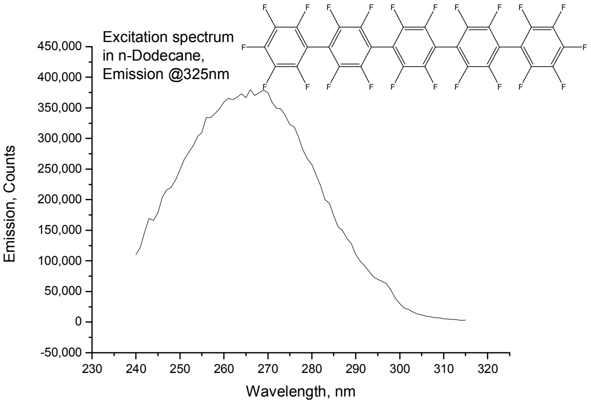

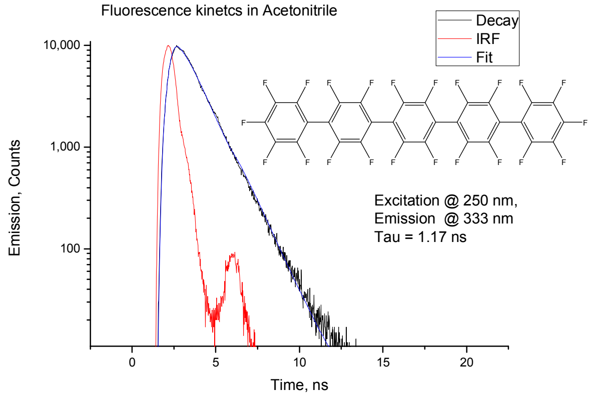

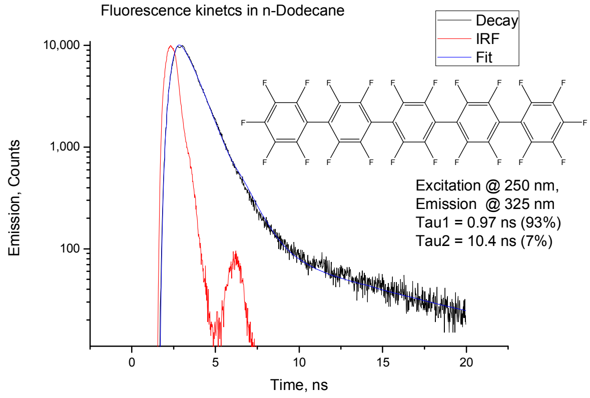

6. Chemical structures and atom numbering systems of perfluoro-1-phenylbenzene (**1**), perfluoro-1',4'-diphenylbenzene (**2**) and dimethylaniline (DMA) for DFT calculations

<table>
<tr><td>1</td><td>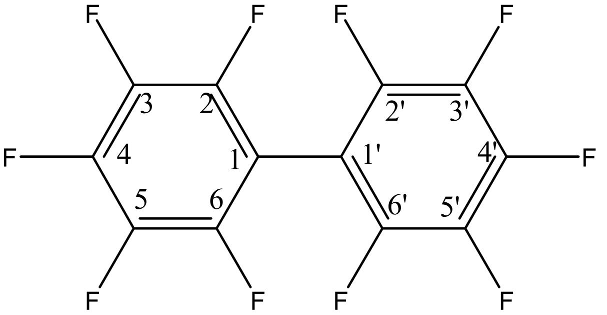</td><td>DMA</td><td>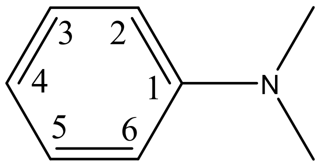</td></tr>
<tr><td>2</td><td colspan="3">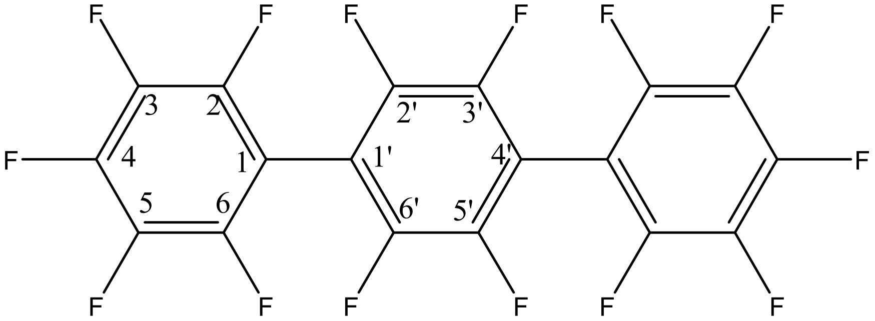</td></tr>
</table>

7. Calculated absorption lines for equilibrium DMA and DMA* structures in heptane (square blue and red points, respectively) and experimental spectra (PhD. Thesis, A.R. Melnikov, 2016, Novosibirsk, Russia) of absorption in *n*-dodecane (blue curves) and luminescence in acetonitrile (red curves)

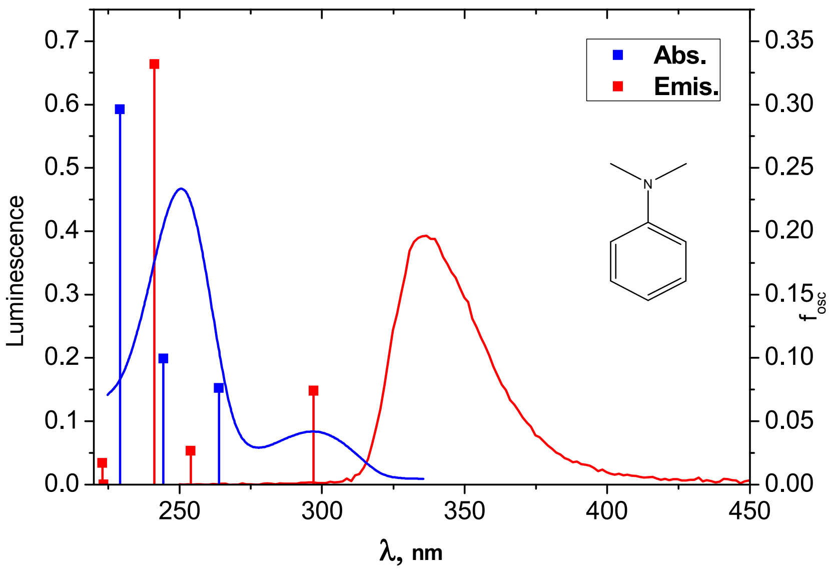

8. Geometric and electronic structures of the needed states for **1**, **2** and DMA

Table S1. Geometric and electronic structures of **1** and **2** in neutral, anionic and first excited states. Total charge, Q; multiplicity, M; bond lengths, $R_{i\text{-}j}$, A; bond angles, $\angle_{i\text{-}j\text{-}k}$, °; dihedral angles, $\angle_{i\text{-}j\text{-}k\text{-}l}$, °; Mulliken charges, q_i; total energy, E_0, eV.

N	Mol.	Q<br>M	$R_{1\text{-}2,\ 1'\text{-}2'}$<br>$R_{2\text{-}3,\ 2'\text{-}3'}$<br>$R_{3\text{-}4,\ 3'\text{-}4'}$<br>$\angle_{2\text{-}1\text{-}1'\text{-}2'}$	$R_{2\text{-}F2,\ 2'\text{-}F2'}$<br>$R_{3\text{-}F3,\ 3'\text{-}F3'}$<br>$R_{4\text{-}F4,\ 1\text{-}1'}$<br>$\angle_{2\text{-}3\text{-}4\text{-}F4}$	$\angle_{6\text{-}1\text{-}2}$<br>$\angle_{1\text{-}2\text{-}3}$<br>$\angle_{2\text{-}3\text{-}4}$<br>$\angle_{3\text{-}4\text{-}5}$	$\angle_{1'\text{-}1\text{-}2}$<br>$\angle_{1\text{-}2\text{-}F2}$<br>$\angle_{2\text{-}3\text{-}F3}$<br>$\angle_{3\text{-}4\text{-}F4}$	q_1, q_2,<br>q_3, q_4,<br>q_{F2}, q_{F3},<br>q_{F4}	$q_{1'}$, $q_{2'}$,<br>$q_{3'}$, $q_{4'}$,<br>$q_{F2'}$, $q_{F3'}$<br>$q_{F4'}$	E_0, eV
1	**1**	0<br>1	1.389, 1.389<br>1.384, 1.384<br>1.386, 1.386<br>68.9	1.330, 1.330<br>1.327, 1.327<br>1.323, 1.481<br>180.0	117.7<br>121.7<br>119.4<br>120.1	121.2<br>119.8<br>120.6<br>119.9	2.03, -0.78<br>0.16, 0.00<br>-0.14, -0.18<br>-0.15	2.03, -0.78<br>0.16, 0.00<br>-0.14, -0.18<br>-0.15	-39608.74
2	**1**•⁻	-1<br>2	1.426, 1.426<br>1.372, 1.372<br>1.395, 1.395<br>43.2	1.348, 1.348<br>1.344, 1.344<br>1.348, 1.430<br>179.7	113.8<br>123.2<br>120.8<br>118.1	123.1<br>119.0<br>120.1<br>120.9	2.29, -0.86<br>0.29, -0.78<br>-0.14, -0.20<br>-0.17	2.29, -0.86<br>0.29, -0.78<br>-0.14, -0.20<br>-0.17	-39610.16
3	**1***	0<br>1	1.402, 1.393<br>1.418, 1.384<br>1.396, 1.387<br>59.4	1.319, 1.331<br>1.330, 1.327<br>1.379, 1.471<br>139.9	113.1<br>123.4<br>122.2<br>113.9	122.7<br>119.0<br>116.7<br>119.8	2.29, -1.00<br>0.18, -0.01<br>-0.09, -0.12<br>-0.08	2.12, -1.09<br>0.33, -0.17<br>-0.13, -0.18<br>-0.15	-39604.01
4	**2**	0<br>1	1.389, 1.388<br>1.385, 1.384<br>1.386, 1.388<br>68.7	1.330, 1.330<br>1.326, 1.330<br>1.323, 1.481<br>180.0	117.7<br>121.6<br>119.4<br>120.1	121.1<br>119.8<br>120.6<br>119.9	1.62, -0.56<br>0.01, 0.17<br>-0.14, -0.17<br>-0.16	1.72, -0.65<br>-0.65, 1.72<br>-0.14, -0.14	-56696.72
5	**2**•⁻	-1<br>2	1.407, 1.421<br>1.381, 1.362<br>1.387, 1.421<br>47.2	1.338, 1.349<br>1.337, 1.349<br>1.337, 1.446<br>180.5	115.0<br>122.8<br>120.2<br>118.9	122.5<br>119.7<br>120.3<br>120.5	1.98, -0.79<br>0.08, 0.10<br>-0.17, -0.19<br>-0.20	1.82, -0.84<br>-0.84, 1.82<br>-0.18, -0.18	-56698.53
6	**2***	0<br>1	1.421, 1.436<br>1.375, 1.357<br>1.394, 1.436<br>42.6	1.327, 1.331<br>1.326, 1.331<br>1.320, 1.425<br>180.0	115.7<br>122.3<br>120.0<br>119.8	122.1<br>119.4<br>120.6<br>120.1	2.23, -0.82<br>0.06, 0.14<br>-0.13, -0.17<br>-0.16	1.91, -0.87<br>-0.87, 1.91<br>-0.12, -0.12	-56692.06

Table S2. Geometric and electronic structure of DMA in neutral and cationic states. Total charge, Q; multiplicity, M; bond lengths, $R_{i\text{-}j}$, A; bond angles, $\angle_{i\text{-}j\text{-}k}$, °; dihedral angles, $\angle_{i\text{-}j\text{-}k\text{-}l}$, °; Mulliken charges, q_i; total energy, E_0, eV.

N	Mol.	Q<br>M	$R_{1\text{-}2}$,<br>$R_{2\text{-}3}$,<br>$R_{3\text{-}4}$	$R_{2\text{-}H2}$,<br>$R_{3\text{-}H3}$,<br>$R_{4\text{-}H4}$	$R_{1\text{-}N}$,<br>$R_{N\text{-}C}$,<br>$R_{C\text{-}H}$	$\angle_{6\text{-}1\text{-}2}$<br>$\angle_{1\text{-}2\text{-}3}$<br>$\angle_{2\text{-}3\text{-}4}$<br>$\angle_{3\text{-}4\text{-}5}$	$\angle_{2\text{-}1\text{-}N}$<br>$\angle_{1\text{-}N\text{-}C}$<br>$\angle_{1\text{-}2\text{-}H2}$<br>$\angle_{2\text{-}1\text{-}N\text{-}C}$	q_1, q_2,<br>q_3, q_4,<br>q_N, q_C	q_{H2},<br>q_{H3},<br>q_{H4}	E_0, eV
1	DMA	0<br>1	1.408<br>1.388<br>1.390	1.081<br>1.085<br>1.083	1.386<br>1.449<br>1.098	117.4<br>120.9<br>121.2<br>118.4	121.3<br>118.7<br>120.5<br>13.4	-0.02, -0.11<br>-0.22, -0.48<br>0.06, -0.42	0.17<br>0.22<br>0.19	-9964.44
2	DMA•⁺	1<br>2	1.428<br>1.372<br>1.401	1.081<br>1.083<br>1.083	1.347<br>1.464<br>1.088	118.8<br>120.0<br>120.5<br>120.3	120.6<br>121.0<br>120.6<br>0.0	-0.19, -0.01<br>-0.18, -0.41<br>0.18, -0.44	0.24<br>0.29<br>0.26	-9958.24

9. Juxtaposition of calculated and experimental emission spectra for single molecule/exciplex for **1**, **2** and DMA

Figures S9.1 and S9.2 show emission spectra for **1** and for complex of **1** with DMA, respectively, calculated for several inter-molecular distances. Comparison of the two figures helps understand which lines in figure S9.2 should be attributed to local emission of **1**. The group of lines in the vicinity of 348 and 289 nm should be assigned to local emission of **1**, as their location does not depend on intermolecular distance. On the other hand, at distances 3.0 and 2.2A new lines appear in the region of 375 and 330 nm. We believe that the appearance of these lines indicates that at close intermolecular distances a new interaction arises that leads to new emissive transitions. A similar picture with even closer resemblance to experimental results can be seen from comparison of figures S9.3 and S9.4 for **2** and DMA. It can further be noted that distance dependencies of oscillator strengths for local and exciplex emission lines shown in Fig. 8 of the main text are very similar for DMA attached to the outer ring of **1** and **2**, with oscillator strengths for local emission (f_l) close to those observed in Figs. S9.1 and S9.3. As exciplex emission line intensity rapidly drops with increasing distance, it is possible that at equilibrium distances the results for intensities of exciplex emission lines with inner and outer assembly will be similar.

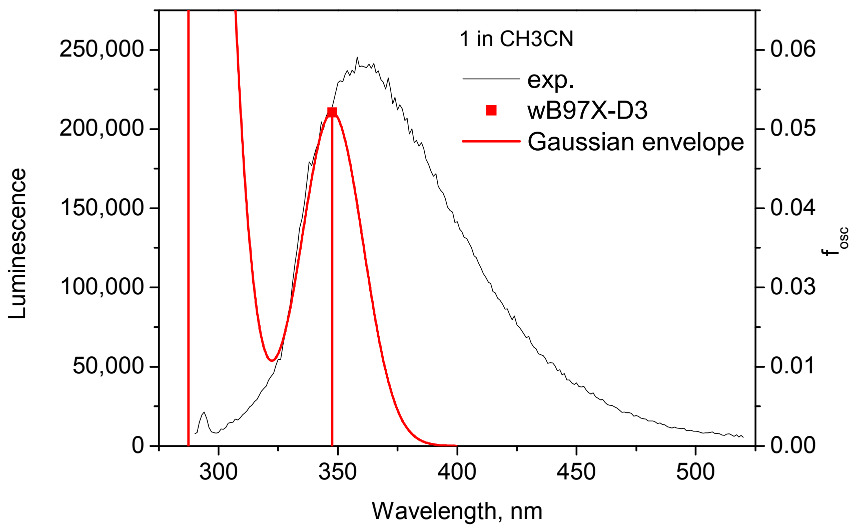

Fig. S9.1. Calculated absorption lines for equilibrium **1***structure in heptane (square points) and experimental luminescence spectrum in acetonitrile.

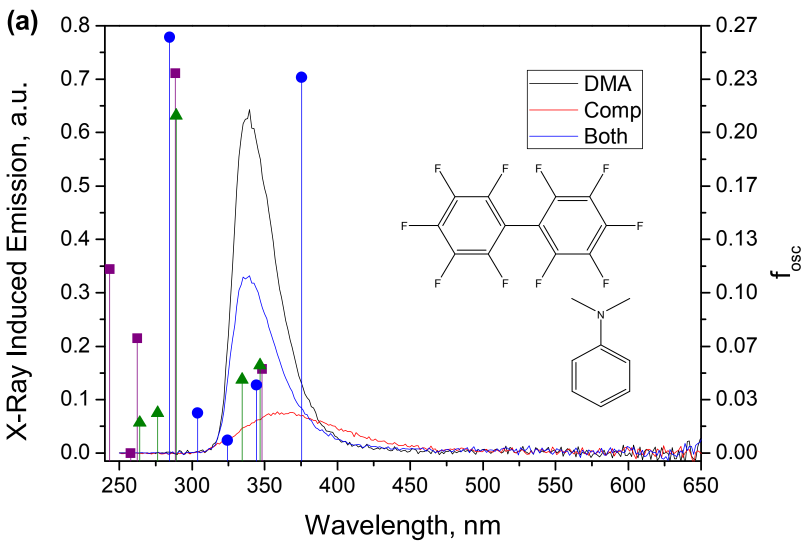

Fig. S9.2. Spectra of X-ray induced luminescence for **1** and/or DMA in degassed *n*-dodecane solution: only DMA (black curve), only **1** (red curve), and their mixture at the same concentrations of components (blue curve). DMA concentration 10^{-2}M, concentrations of **1** 5•10^{-3}M. Calculated spectra of **1** with DMA attached to **outer** ring at the following distances: 10A (purple square points), 3.0A (green triangle points), 2.2A (blue circle points).

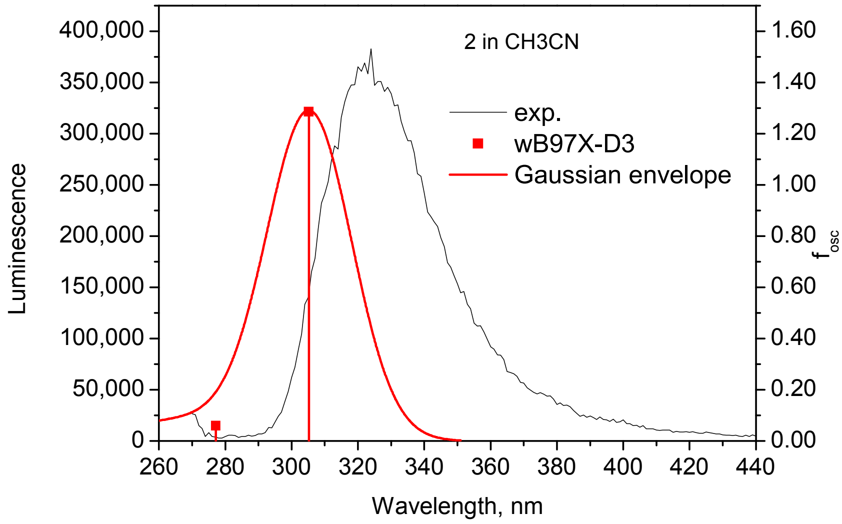

Fig. S9.3. Calculated absorption lines for equilibrium **2*** structure in heptane (square points) and experimental luminescence spectrum in acetonitrile.

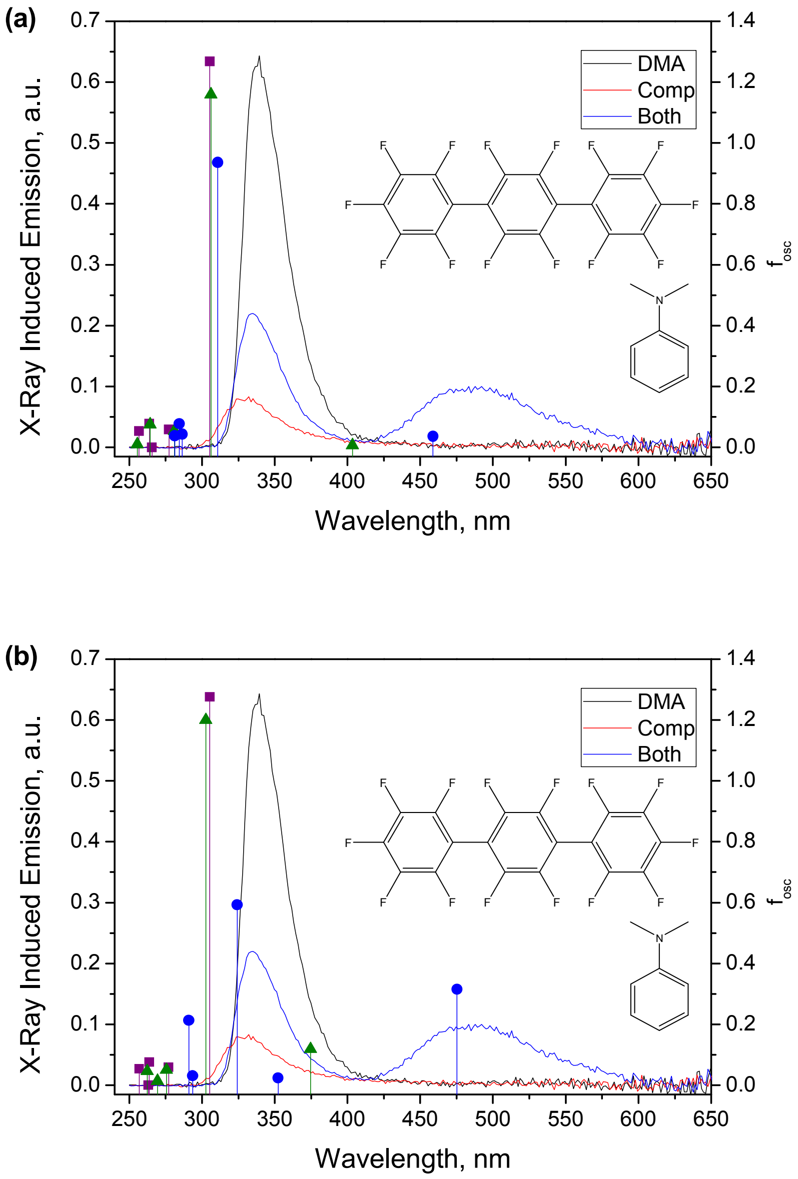

Fig. S9.4. Spectra of X-ray induced luminescence for **2** and/or DMA in degassed *n*-dodecane solution: only DMA (black curve), only **2** (red curve), and their mixture at the same concentrations of components (blue curve). DMA concentration 10^{-2}M, concentrations of **2** $1.8 \cdot 10^{-3}$M. Calculated spectra of **2** with DMA attached to **inner (a) and outer (b)** rings at the following distances: 10A (magenta square points), 3.0A (green triangle points), 2.2A (blue circle points).
